# Lifestyle behaviors associated with the initiation of renal replacement therapy in Japanese patients with chronic kidney disease: a retrospective cohort study using a claims database linked with specific health checkup results

**DOI:** 10.1186/s12199-021-01022-3

**Published:** 2021-10-09

**Authors:** Azusa Hara, Takumi Hirata, Tomonori Okamura, Shinya Kimura, Hisashi Urushihara

**Affiliations:** 1 Division of Drug Development and Regulatory Science, Faculty of Pharmacy, Keio University, 1-5-30, Shibakoen, Minato-ku, Tokyo, 105-8512 Japan; 2 Department of Public Health, Hokkaido University Faculty of Medicine, Sapporo, Japan; 3 Department of Preventive Medicine and Public Health, Keio University School of Medicine, Tokyo, Japan; 4 JMDC Inc., Tokyo, Japan

**Keywords:** Chronic kidney disease, Lifestyle behaviors, Renal replacement therapy, Japanese workers, Database

## Abstract

**Background:**

Chronic kidney disease (CKD) is an independent risk factor for progression to an end-stage renal disease requiring dialysis or kidney transplantation. We investigated the association of lifestyle behaviors with the initiation of renal replacement therapy (RRT) among CKD patients using an employment-based health insurance claims database linked with specific health checkup (SHC) data.

**Methods:**

This retrospective cohort study included 149,620 CKD patients aged 40–74 years who underwent a SHC between April 2008 and March 2016. CKD patients were identified using ICD-10 diagnostic codes and SHC results. We investigated lifestyle behaviors recorded at SHC. Initiation of RRT was defined by medical procedure claims. Lifestyle behaviors related to the initiation of RRT were identified using a Cox proportional hazards regression model with recency-weighted cumulative exposure as a time-dependent covariate.

**Results:**

During 384,042 patient-years of follow-up by the end of March 2016, 295 dialysis and no kidney transplantation cases were identified. Current smoking (hazard ratio: 1.87, 95% confidence interval, 1.04─3.36), skipping breakfast (4.80, 1.98─11.62), and taking sufficient rest along with sleep (2.09, 1.14─3.85) were associated with the initiation of RRT.

**Conclusions:**

Among CKD patients, the lifestyle behaviors of smoking, skipping breakfast, and sufficient rest along with sleep were independently associated with the initiation of RRT. Our study strengthens the importance of monitoring lifestyle behaviors to delay the progression of mild CKD to RRT in the Japanese working generation. A substantial portion of subjects had missing data for eGFR and drinking frequency, warranting verification of these results in prospective studies.

**Supplementary Information:**

The online version contains supplementary material available at 10.1186/s12199-021-01022-3.

## Background

Chronic kidney disease (CKD), diagnosed by a gradual reduction in kidney function or renal dysfunction for more than three months, is a significant public health issue worldwide [1]. In Japan, approximately 13.3 million people are considered to have CKD, accounting for one-eighth of the Japanese adult population [2]. CKD is an independent risk factor for progression to end-stage renal disease requiring dialysis or kidney transplantation [3, 4]. In 2017, approximately 335,000 patients were receiving maintenance dialysis, [5] the medical cost of which reached 1600 billion yen and accounted for 4% of the total health care budget in Japan that year [6]. In contrast, far fewer kidney transplantations were performed that year, in approximately 1700 patients [7].

Lifestyle-related diseases, such as diabetes, hypertension, and dyslipidemia, are known risk factors for the prevalence and incidence of developing CKD [3, 8]. Healthy lifestyle behaviors can prevent lifestyle-related diseases and consequently avoid the onset and progression of CKD. However, due to difficulties in ensuring sufficient statistical power to detect and analyze low-incidence RRT events, few studies have investigated factors which predict the initiation of chronic renal replacement therapy (RRT) among CKD patients, including lifestyle factors [9].

“Specific Health Checkups (SHC) and Specific Health Guidance” commenced in 2008 as part of the national health insurance system in Japan [10]. Employers are mandated to provide the insured and their dependents aged 40–74 years with the opportunity to take SHC annually for the early detection of metabolic syndrome, a common lifestyle-related disease [11–13].

Here, using an employment-based large-scale claims database linked with SHC data, we investigated the association of cumulative exposure to lifestyle behaviors with the initiation of chronic RRT among CKD patients identified using both claims records and SHC results.

## Methods

### Data source

This retrospective cohort study was performed using a large-scale claims database linked with the results of SHC items, consisting of laboratory measurements, medical interviews on lifestyle diseases, and a self-administrated questionnaire on lifestyle behaviors. The claims records and linked SHC data were provided by multiple employment-based health insurance plans to JMDC Inc. (Tokyo, Japan). Details of lifestyle behaviors and lifestyle disease profiles of the population in the JMDC database have been described elsewhere [13, 14]. The JMDC database included 1,450,215 enrollee plans as of March 31, 2016, consisting of employees and their dependents covered by company-run health insurance. We used the claims records and SHC results between April 2008 and March 2016.

### Study subjects

**Fig. 1:**
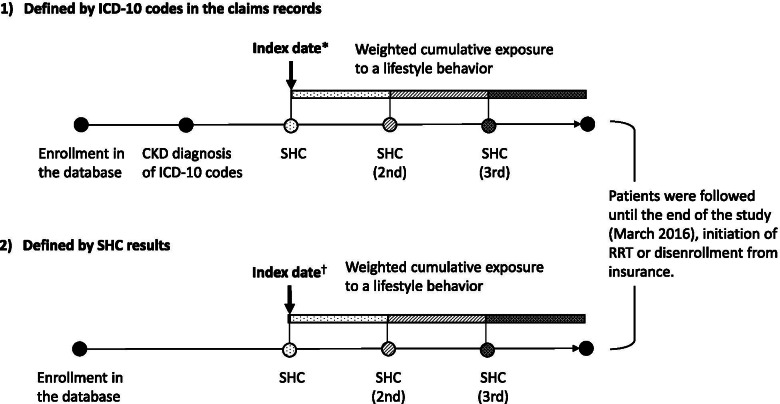
Identification criteria for CKD patients and definition of index date. CKD chronic kidney disease; ICD-10 International Classification of Diseases, Tenth Revision; RRT renal replacement therapy; SHC Specific Health Checkups. (1) Index date*: The first date of SHC after the initial diagnosis of CKD in the claims records. (2) Index date^†^: The first date when they met the CKD criteria according to the SHC results. CKD patients were identified by meeting either or both of the following criteria: (1) having the disease codes for CKD coded by ICD-10 as shown in eTable 1 recorded in the claims database and (2) having an estimated glomerular filtration rate less than 60 ml/min per 1.73 m^2^ or positive proteinuria according to the SHC results

We studied CKD subjects aged 40 to 74 years who underwent SHC during the study period. CKD patients were identified by either or both of the following criteria: (1) a disease code for CKD according to the International Classification of Diseases, Tenth Revision (ICD-10), [15] as shown in eTable 1, recorded in the claims database; and (2) an estimated glomerular filtration rate (eGFR) less than 60 ml/min per 1.73 m^2^ or positive proteinuria according to the SHC results [3]. The patients were followed from the index date, defined as the first date of SHC after the initial diagnosis of CKD in the claims records or the first date on which they met the second CKD criterion above, whichever came earlier (Fig. 1). In addition, CKD patients eligible for the study had to have a look back period to ascertain the absence of chronic RRT between enrollment in the database and the index date. Advanced cancer patients with the ICD-10 codes of C00─D48 and cancer therapy recorded in the claims database were excluded (eTables 2.1 and 2.2). Patients who were disenrolled from their insurance in the same month as the index date were also excluded. Participants were followed until the end of the study period, or the date they initiated RRT or were disenrolled from insurance, which came earlier.

### Study variables

The primary study outcome was chronic RRT, including dialysis or kidney transplantation, and was determined by the medical procedure codes in the claims records (eTable 3) [16]. Dialysis was considered positive when claims were present for both “outpatient medical management fees for chronic maintenance dialysis patients (B001)” and procedures related to dialysis (C102, C102-2, J038, J042) in the same month. The former code was adopted to identify chronic RRT and exclude temporary dialysis aimed at treating acute renal failure (eTable 3). Kidney transplantation was considered positive when claims were present for procedures concerning cadaveric renal transplantation (K780) or living kidney transplantation (K780-2) (eTable 3). We evaluated the first event of chronic RRT after the index date.

Data for body mass index, serum creatinine, and proteinuria were collected by physical measurement and biochemical examination at SHC. eGFR was calculated using serum creatinine level and the following formula for Japanese patients: eGFR (ml/min per 1.73 m^2^)=194×(serum creatinine)^-1.094^×age^-0.287^ (multiply by 0.739 in case of woman) [17]. CKD patients were classified into the categories of the Kidney Disease: Improving Global Outcomes (KDIGO) classification 2012 which was based on disease, eGFR category (G1 to G5) and albuminuria category (A1 to A3) [18]. Since proteinuria in this study was detected by dipstick at SHC, we substituted a result of dipstick test for albuminuria level to classify patients into the albuminuria category [3]. We also classified the CKD patients into the four risk stages in combination of eGFR category and albuminuria category [18]. We investigated the 11-item survey results on lifestyle behaviors using the self-administered questionnaire of the annual SHC, including current smoking, regular exercise, regular walking, walking fast, skipping breakfast, eating speed, eating dinner late, late-evening snacking, frequency and amount of drinking alcohol, and taking sufficient rest along with sleep (eTable 4) [13]. Histories of cardio- and cerebrovascular disease were defined by ICD-10 codes in the claims data (eTable 5), and medications for diabetes, hypertension, and hypercholesterolemia were determined by drug classification code 87 in the Japan Standard Commodity Classification in claims records (eTable 6).

### Statistical analysis

Baseline characteristics and lifestyle behaviors at the index date were summarized using descriptive statistics and proportions.

Cox proportional hazards regression models were constructed to estimate hazard ratios (HRs) of the initiation of RRT and included lifestyle behaviors as explanatory variables. Cumulative exposure to a lifestyle behavior for each individual was estimated using a recency-weighted cumulative exposure model: [19]

$${E}_1={E}_{visit\ 1}$$

$${E}_{n\ \left(n\ge 2\right)}=\frac{E_{visit\ 1}}{2^{n-1}}+{\sum}_{k=2}^n\frac{E_{visit\ k}}{2^{n+1-k}}$$

where *E*_*n*_ denotes estimated recency-weighted cumulative exposure at visit *n*, and *E*_*visit n*_ denotes exposure at visit *n*.

We did not adjust for the self-reported amount of daily alcohol intake, although the SHC does have a survey item asking about alcohol intake, including both daily amount and weekly frequency. For 58.5% of respondents who answered “rare” for frequency, the amount of alcohol intake at baseline was missing, indicating that the validity of data for the amount of alcohol intake was suboptimal. We therefore adjusted only for weekly frequency, but not for daily amount of alcohol consumption. The other covariates in the Cox model included age; gender; body mass index; eGFR; proteinuria; medications for diabetes, hypertension, and hypercholesterolemia; histories of cardiovascular disease and cerebrovascular disease; and the total number of checkups taken. All covariates were included in the model as time-dependent covariates (except for gender). Multicollinearity was assessed with Spearman’s rank correlation coefficients and the variance inflation factor (VIF) values.

Sensitivity analyses were performed using CKD patients defined by either of the criteria mentioned earlier, namely either ICD-10 code (criteria 1, eTable 1) or SHC data (criteria 2). To adjust for confounding by diabetes, hypertension, and hypercholesterolemia, the Cox hazard model in the primary analysis included the use of medications for these diseases as covariates. We added systolic blood pressure, LDL cholesterol, and HbA1c as time-dependent covariates in the Cox regression model for a post hoc analysis.

Analyses were conducted using SAS version 9.4 for Windows (SAS Institute Inc., Cary, NC, USA).

### Ethical considerations

The study protocol was approved by the Keio University Faculty of Pharmacy ethics committee for research involving humans (No. 190509-2). Consistent with local ethical guidelines for medical research involving human subjects, [20] the requirement that study participants provide informed consent was waived.

## Results

### Characteristics at baseline

**Fig. 2:**
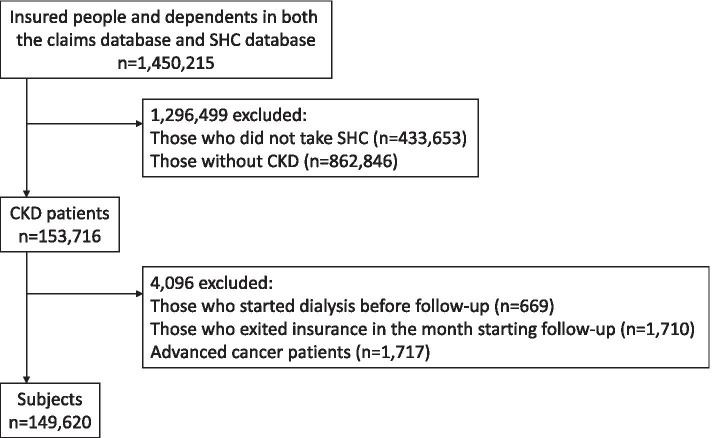
Flow chart of subject selection. CKD chronic kidney disease, SHC specific health checkups

Among 1,450,215 enrollees, 153,716 CKD patients with SHC data were extracted. We excluded 4096 patients who had RRT during the look-back period (*n*=669), were disenrolled from their insurance in the month of the index date (*n*=1710), and had advanced cancer (*n*=1717). Finally, 149,620 patients were analyzed in the present study (Fig. 2). During a total follow-up of 384,042 patient-years among 149,620 patients (median follow-up of 2.3 years), 295 dialysis cases and no kidney transplantation cases were identified, amounting to an incidence of 0.77 per 1000 patient-years. At the time when RRT was initiated, the number of CKD patients having ICD-10 codes related to glomerulonephritis was the most common (*n*=130; ICD-10 codes of N02, N03, N04, N05, N06, and N391), followed by diabetic nephropathy (*n*=98; E102, E112, E132, and E142) and nephrosclerosis (*n*=23; I12 and N26). There were no CKD patients who had the hereditary renal disease (N07 and E851).

At baseline, cases had lower eGFR than non-cases (15 [interquartile range (IQR) 8, 33] ml/min/1.73 m^2^ *vs* 59 [IQR 56, 74] ml/min/1.73 m^2^), and a higher prevalence of proteinuria (78.6% *vs* 40.4%) and a diagnosis of CKD (79.3% *vs* 29.9%) (Table 1). Cases also had a higher prevalence of high-risk category by KDIGO CKD classification (eTable 7), [18] a history of coronary artery disease and cerebrovascular disease, and medications for hypertension, diabetes, and hypercholesterolemia (Table 1). Further, cases had an approximately 10% lower proportion of female, regular walking, and walking fast and less frequent alcohol drinking than non-cases at baseline (Table 2). The prevalence between the cases and non-cases of the other lifestyle behaviors were similar (Table 2).

**Table 1:** Baseline characteristics

	Initiation of renal replacement therapy	
	(–)	(+)
Number of subjects	149,325	295
Follow-up time, years, median [IQR]	2.3 [1.2, 3.4]	1.9 [1.0, 3.0]
Age, years, median [IQR]	52 [46, 59]	51 [46, 56]
Type of insurance, *n* (%)		
Insured workers	119,762 (80.2)	274 (92.9)
Dependents	29,563 (19.8)	21 (7.1)
Females, *n* (%)	44,372 (29.7)	34 (11.5)
BMI, kg/m^2^, mean (SD)	24.0 (4.1)	24.9 (4.9)
eGFR, ml/min/1.73 m^2^, median [IQR]	59 [56, 74]	15 [8, 33]
eGFR<60 ml/min/1.73 m^2^, *n* (%)	53,858 (36.1)	98 (33.2)
Proteinuria (1+ or more, dipstick), *n* (%)	60,318 (40.4)	232 (78.6)
Diagnosis of CKD, *n* (%)	44,701 (29.9)	234 (79.3)
Medication for diabetes, *n* (%)	17,804 (11.9)	113 (38.3)
Medication for hypertension, *n* (%)	45,669 (30.6)	263 (89.2)
Medication for hypercholesterolemia, *n* (%)	30,428 (20.4)	147 (49.8)
History of cardiovascular disease, *n* (%)	21,571 (14.4)	115 (39.0)
History of cerebrovascular disease, *n* (%)	8156 (5.5)	34 (11.5)
Number of checkups received, median [IQR]	2 [1, 4]	2 [1, 3]

*BMI* body mass index, *CKD* chronic kidney disease, *eGFR* estimated glomerular filtration rate, *IQR* interquartile range, *SD* standard difference

Data are presented as the number of subjects (%), median [IQR], or mean (SD)

Missing values were included in the calculation of prevalence of patients who had lower eGFR (<60 ml/min/1.73 m^2^) and proteinuria

**Table 2:** Lifestyle behaviors at baseline

	Initiation of renal replacement therapy	
	(–)	(+)
Number of subjects	149,325	295
Current smoking, *n* (%)	38,484 (25.8)	82 (27.8)
Regular exercise, *n* (%)	32,493 (21.8)	45 (15.3)
Regular walking, *n* (%)	40,067 (26.8)	49 (16.6)
Walking fast, *n* (%)	53,668 (35.9)	57 (19.3)
Dietary habits, *n* (%)		
Frequent skipping breakfast	18,177 (12.2)	35 (11.9)
Eating speed		
Slow	10,669 (7.1)	13 (4.4)
Normal	68,335 (45.8)	124 (42.0)
Fast	37,653 (25.2)	69 (23.4)
Eating dinner late	35,510 (23.8)	85 (28.8)
Frequent late-evening snacking	19,323 (12.9)	30 (10.2)
Alcohol drinking frequency, *n* (%)		
Rare	54,207 (36.3)	137 (46.4)
Occasional	41,629 (27.9)	76 (25.8)
Daily	35,143 (23.5)	38 (12.9)
Sufficient rest along with sleep, *n* (%)	74,564 (49.9)	136 (46.1)

Data are presented as the number of subjects (%)

Missing values were included in the calculation of the prevalence for each lifestyle behavior

Among all baseline variables, the most common missing value was eGFR, at 65.4% in the cases and 38.8% in the non-cases (eTable 8). The second most common missing value at baseline was the amount of alcohol consumption. The alcohol consumption was missing in 44.7% of the cases and 38.4% of the non-cases (eTable 8). Missing observations for lifestyle behavior items ranged from 6.2 to 44.7% of all patients (eTable 8). The amount of alcohol consumption at baseline was missing in 58.5%, 11.0%, and 9.1% of respondents who answered “rare,” “occasional,” and “everyday,” respectively, to the item on alcohol intake frequency.

Patients with missing eGFR values had a higher prevalence of proteinuria (59.6% *vs* 28.3%) and diagnosis of CKD (44.8% *vs* 20.7%) than those with eGFR values (eTable 9). Lifestyle behaviors were similar between patients with and without missing eGFR values (eTable 10).

### Association of daily lifestyle behaviors with the initiation of chronic RRT

60,481 patients with eGFR values were eligible for multivariate Cox hazards regression analysis for HRs of the initiation of RRT (Table 3). 68 RRT cases were included in the analysis. Among the survey items of lifestyle behaviors, current smoking (HR 1.87, 95% confidence interval [CI] 1.04─3.36), frequent breakfast-skipping (HR 4.80, 95% CI 1.98─11.62), and sufficient rest along with sleep (HR 2.09, 95% CI 1.14─3.85) were significantly associated with the initiation of RRT (Table 3). Female gender (HR 0.20, 95% CI 0.05─0.72), higher eGFR level (HR 0.81, 95% CI 0.78─0.84), and number of SHCs taken (HR 0.55, 95% CI 0.38─0.80) were significantly associated with decreased risk for the initiation of RRT, while antidiabetic medication (HR 2.65, 95% CI 1.49─4.70) was associated with increased risk (Table 3).

**Table 3:** Hazard ratios for the initiation of renal replacement therapy (*n*=60,481)

	HR	95%CI
Age (per 1-year increase)	0.99	(0.95–1.03)
Women	0.20	(0.05–0.72)
BMI (per 1 kg/m^2^)	0.95	(0.88–1.03)
eGFR (per 1-ml/min/1.73 m^2^)	0.81	(0.78–0.84)
Proteinuria	2.65	(0.79–8.90)
Medication for diabetes	2.65	(1.49–4.70)
Medication for hypertension	3.82	(0.67–21.65)
Medication for hypercholesterolemia	0.93	(0.53–1.61)
History of cardiovascular disease	1.22	(0.69–2.15)
History of cerebrovascular disease	0.60	(0.26–1.34)
Number of checkups received (per 1-time)	0.55	(0.38–0.80)
Current smoking	1.87	(1.04–3.36)
Regular exercise	1.05	(0.52–2.14)
Regular walking	1.00	(0.51–1.93)
Walking fast	1.24	(0.60–2.59)
Dietary habits		
Frequent skipping breakfast	4.80	(1.98–11.62)
Eating speed		
Slow	1.00	[Reference]
Normal	1.49	(0.52–4.30)
Fast	2.13	(0.69–6.59)
Eating dinner late	0.55	(0.29–1.02)
Frequent late-evening snacking	1.71	(0.50–5.94)
Alcohol drinking frequency		
Rare	1.00	[Reference]
Occasional	1.10	(0.60–2.05)
Daily	0.46	(0.17–1.28)
Sufficient rest along with sleep	2.09	(1.14–3.85)

*CI* confidence interval, *HR* hazard ratio, *N/A* not applicable

Spearman’s rank correlation analysis did not indicate a strong correlation (>0.70) among covariates, with the highest being 0.50 between proteinuria and eGFR. The VIF values of all variables were less than 10 (data not shown).

### Sensitivity analyses

Among the population of patients with CKD (*n*=15,389) whose definition was based on ICD-10 codes only, 67 cases of RRT were identified. Current smoking (HR 2.21, 95% CI 1.28─3.80), frequent breakfast-skipping (HR 4.88, 95% CI 1.79─13.35), and sufficient rest along with sleep (HR 2.08, 95% CI 1.10─3.93) were significantly associated with an elevated risk of the initiation of RRT. Among 51,931 patients with CKD whose definition was based on lab test results of SHC only, 65 cases were identified. Frequent breakfast-skipping (HR 5.55, 95% CI 2.24─13.72) was significantly associated with RRT initiation and current smoking (HR 1.72, 95% CI 0.97─3.05), whereas sufficient rest along with sleep (HR 1.97, 95% CI 0.99─3.92) was not significantly associated with RRT initiation. HRs for the other lifestyle behaviors for both groups were similar to the results of the primary analysis (data not shown).

When systolic blood pressure, LDL cholesterol, and HbA1c were included in the Cox hazards model, similar HRs to the primary analysis were obtained for current smoking (HR 1.80, 95% CI 0.87─3.73), frequent breakfast-skipping (HR 3.49, 95% CI 1.35─9.00), and sufficient rest along with sleep (HR 2.11, 95% CI 1.01─4.43).

## Discussion

To our knowledge, this is the first study to examine the association of lifestyle behaviors and initiation of chronic RRT in CKD patients using a large claims database linked with SHC data. Lifestyle behaviors of smoking, skipping breakfast, and sufficient rest along with sleep were independently associated with the initiation of RRT.

At baseline, cases had a lower eGFR and a higher prevalence of proteinuria and history of cardio-cerebrovascular diseases than the non-cases by definition. It is apparent that the cases already had decreased renal function and had been in an unhealthy condition at baseline. On the other hand, lifestyle behaviors between the cases and non-cases were similar, except for walking fast and frequency of alcohol drinking. The healthy lifestyle behaviors observed in the cases may imply either or both good compliance with physician guidance on the prevention or treatment of disease, and the voluntary adoption of health-conscious behavior. A strengthening of advice and motivation from occupational doctors might make employees aware of the necessity of improving their health condition and induce them to take action to change their lifestyle to avoid RRT initiation.

Smoking had a significant association with the initiation of RRT. Our present results from a large claims database linked with SHC data are consistent with previous findings from a meta-analysis and several cohort studies that indicated that smoking was a risk factor for CKD progression [21–23]. In 1951 CKD patients from a cohort study in Korea, the hazard ratio for adverse kidney outcome was attenuated as the duration of smoking cessation increased [24]. Smoking could be a modifiable factor in delaying CKD progression, given that the 2018 Evidence-based Clinical Practice Guideline for CKD recommends smoking cessation for CKD patients [3].

Skipping breakfast was associated with the initiation of RRT in the present study. Associations of skipping breakfast with weight gain, insulin resistance and type 2 diabetes have been suggested [25–27]. A sensation of hunger by skipping breakfast may promote overeating later in the day, [28–30] and an overactivity in the hypothalamic-pituitary-adrenal axis. This prolonged fasting is reported to cause increased blood pressure [31]. Metabolic syndrome and its components, including abdominal obesity, dyslipidemia, elevated fasting glucose, and high blood pressure, were associated with the progression of CKD [32]. Habitually skipping breakfast is likely to lead to metabolic syndrome components and aggravate renal function. Our results emphasize the importance of strengthening guidance on dietary habits for CKD patients [33].

It may be reasonable to ascribe the positive association between the answer “taking sufficient rest along with sleep” and the initiation of RRT to reverse causality. The SHC survey item which asked about rest and sleep did not specify a definition for “taking sufficient rest along with sleep”. Most subjects in the present study were working generation and seemed to be busy with their regular work. They might therefore have perceived that they received insufficient rest and sleep. In contrast, unhealthy subjects may have answered “yes” to the question even though underlying health conditions might have required them to take longer rest and sleep. Because of its ambiguity, this survey item does seem to be a good indicator of the quality of rest. Further, the answer to this item seems to be based on subjective perception. Therefore, it may potentially cause a bias towards both directions. A conclusive answer to this question requires prospective studies with well-designed questionnaires to explore the association between rest and initiation of RRT.

Proteinuria was found not to be an independent predictor of initiation of RRT, despite many consistent reports that increasing proteinuria is associated with the risk of CKD progression [34, 35]. Patients who have been diagnosed with CKD and started medical care were no longer required to take an annual SHC, and their lab tests were therefore conducted during their regular hospital visits. Because the study database does not contain the lab results from regular visits, our analysis cannot consider those patients with missing SHC test data, who likely have impaired kidney function, and our results might consequently be applicable only to those patients with mild severity. Short follow-up duration might be another reason for the failure to detect an association between proteinuria and initiation of RRT. In addition, confirmation of a diagnosis of kidney dysfunction would typically require more than a single event of proteinuria at baseline.

CKD in the present study was primarily defined using both the ICD-10 code in the claims database and the SHC results to optimize validity. The ICD-10 codes of CKD in the claims database are likely to include the false-positive CKD cases because of the need to ensure reimbursement for lab tests. On the other hand, a single, annual SHC result in this study by itself may not detect patients with a persistently low eGFR level (less than 60 ml/min per 1.73 m^2^) of more than 3 months’ duration, which is the standard definition of CKD in clinical practice [3]. A previous validation study conducted at an acute care hospital in Australia reported that the sensitivity and specificity of ICD-10 codes for identifying CKD cases were 54.1% and 90.2%, respectively [36]. Our definition of CKD likely resulted in fewer false-negative cases, because we identified cases using either or both the ICD-10 codes and eGFR values and in turn likely provided better sensitivity than that in the previous study [36].

The strength of this study is its large sample size and long-term follow-up obtained using a large-scale database which included both claims and checkup records, and which consequently provided sufficient power to detect a small number of events. The incidence of RRT is not high among our study population; however, we believe that improving lifestyle factors is an achievable goal which is potentially associated with reducing the risk of RRT initiation among the Japanese patients with mild CKD. In addition, dialysis is time-intensive and expensive and requires dietary restrictions [37]. Dialysis patients have been reported as having low quality of life [38]. Our findings would contribute to better management of patients with mild kidney impairment. In addition, we employed recency-weighted cumulative exposure as a time-dependent covariate to model the cumulative effects of lifestyle behaviors, as similarly used in a previous study, [19] and consider that this analytical method is suitable for assessing the cumulative, long-term effects of lifestyle behaviors.

### Limitations

It is worth mentioning several possible limitations in interpreting our study results. First, the possibility of selection bias needs to be considered when generalizing the present findings. The JMDC database included individuals aged up to 75 years old, and our findings might not therefore be generalizable to the elderly population. A previous study of participants receiving regular health checkups in Japan reported that the prevalence of eGFR<60 ml/min/1.73m^2^ increased linearly as age advanced from the 20 to 80s and over, from 0.1 to 44.6% in males, and 0.2 to 46.1% in females [2]. Our participants were a working-age population, and likely to have less confounding factors for renal impairment such as aging and comorbidities than an elderly population, as shown by the low incidence of RRT [8]. We therefore consider that the association between the effects of lifestyle behaviors on renal function in our study population were less complicated and that any associations would likely be detected in a more straightforward manner. On the other hand, the possibility of a healthy worker effect cannot be denied. The study subjects in the present study were the employees and dependents of mid-sized and large companies in secondary and tertiary industries which had a sufficiently large financial size and strength to run their own health insurance plans [13]. Missing data are another source of bias which should be considered when interpreting the results and generalizability of the study. The complete dataset of patients without missing covariates was used for the Cox hazard models in the present study. The proportion of missing eGFR values was high, especially among cases, probably because the measurement of serum creatinine was performed at the discretion of payers at the SHC, and most patients with CKD likely had serum creatinine measurements in their regular hospital visits, not in SHC [39]. Missing eGFR values and urine testing appeared “not at random,” and therefore likely resulted in biased estimates, as discussed above.

Second, we did not fully adjust for the potential effects of drugs on kidney function. Acute kidney injury is common in cancer patients at risk for infection, sepsis, tumor lysis syndrome, drug-associated toxicities, and other comorbidities that significantly increase the risk of acute kidney injury [40]. Chemotherapy and a severely ill condition are strong confounding factors in the association between renal dysfunction and lifestyle behaviors. To minimize potential confounding by chemotherapies and cancer, we excluded patients with advanced cancer. Some other medicines also cause renal disorders, including NSAIDs and antimicrobials, [41] but the use of these medicines was not adjusted for in the analyses because the use of over-the-counter NSAIDs was not captured in the claims database, and because of the difficulty of accurately adjusting for the impact of various kinds of antimicrobial agents, usually used in the short term, on renal function.

Third, the self-reported lifestyle assessment may have included reporting bias. In addition, a degree of bias caused by missing data in the self-administrated questionnaire was unavoidable.

There is also a potential risk of misclassification bias since covariates of comorbidity and concomitant medication were defined using claims information. Although we did not perform a validation study of claims diagnosis codes for cardiovascular and cerebrovascular diseases, several studies assessing the validity of ICD-10 codes to identify cardiovascular and cerebrovascular diseases from the Japanese claims database reported reasonable PPVs for those outcomes [42–44]. A previous study showed the high sensitivity of the information on medication use collected from the nationwide electronic pharmacy records with medication-containing blood samples among patients at a Danish university hospital (sensitivity=0.93) [45]. Claims data is reported to be a useful tool to capture the regular medication users [46].

Finally, given the nature of retrospective evaluation using a secondary database, potential bias due to unmeasured or unknown confounders is unavoidable. A well-designed prospective study which considers all possible factors associated with the initiation of chronic RRT is warranted.

## Conclusions

Among CKD patients, lifestyle behaviors of smoking, skipping breakfast, and sufficient rest along with sleep were independently associated with the initiation of chronic RRT. Our study strengthens the importance of monitoring lifestyle behaviors to delay the progression of mild CKD to RRT in the Japanese working generation. A substantial portion of subjects had missing data for eGFR and drinking frequency, warranting verification of these results in prospective studies.

## Supplementary Information

**Additional file 1: eTable 1**. Diagnosis codes for chronic kidney disease. **eTable 2**. Codes in relation to cancer and cancer therapy. **eTable 2.1**. Diagnosis codes for cancer. **eTable 2.2**. Therapeutic category codes for cancer therapy. **eTable 3**. Medical procedure codes related to the initiation of renal replacement therapy. **eTable 4**. Details of lifestyle behaviors. **eTable 5**. Diagnosis codes related to cardio- and cerebrovascular diseases. **eTable 6**. Therapeutic category codes for drugs on diabetes, hypertension, and dyslipidemia. **eTable 7**. Kidney Disease: Improving Global Outcomes (KDIGO) CKD Classification at baseline. **eTable 8**. Prevalences of missing value in each variable. **eTable 9**. Baseline characteristics of patients with or without missing of eGFR. **eTable 10**. Baseline lifestyle behaivors of patients with or without missing of eGFR.

## Data Availability

The datasets for the study are not publicly available due to the data license agreement with Japan Medical Data Center Inc. Data are, however, available from the corresponding author upon reasonable request and with the permission of the Japan Medical Data Center Inc.

## Abbreviations

CKD: Chronic kidney disease
RRT: Renal replacement therapy
SHC: Specific health checkup
ICD-10: International Classification of Diseases, Tenth Revision
IQR: Interquartile range
eGFR: Estimated glomerular filtration rate
HR: Hazard ratio
VIF: Variance inflation factor
KDIGO: Kidney Disease: Improving Global Outcomes
