# Supplementary material for: Lifestyle behaviors associated with the initiation of renal replacement therapy in Japanese patients with chronic kidney disease: a retrospective cohort study using a claims database linked with specific health checkup results
Source: Environ Health Prev Med. 2021 Oct 9;26:102. doi: 10.1186/s12199-021-01022-3 (PMC8502396; doi:10.1186/s12199-021-01022-3)
Supplement: Supplementary file 1 — Additional file 1: eTable 1. Diagnosis codes for chronic kidney disease. eTable 2. Codes in relation to cancer and cancer therapy. eTable 2.1. Diagnosis codes for cancer. eTable 2.2. Therapeutic category codes for cancer therapy. eTable 3. Medical procedure codes related to the initiation of renal replacement therapy. eTable 4. Details of lifestyle behaviors. eTable 5. Diagnosis codes related to cardio- and cerebrovascular diseases. eTable 6. Therapeutic category codes for drugs on diabetes, hypertension, and dyslipidemia. eTable 7. Kidney Disease: Improving Global Outcomes (KDIGO) CKD Classification at baseline. eTable 8. Prevalences of missing value in each variable. eTable 9. Baseline characteristics of patients with or without missing of eGFR. eTable 10. Baseline lifestyle behaivors of patients with or without missing of eGFR. [file 12199_2021_1022_MOESM1_ESM.pdf]

EHPM

Data Supplement

This Data Supplement has been provided by the authors to give readers additional information about their work.

Supplement to: Hara A, *et al.*, *Lifestyle Behaviors Associated with the Initiation of Renal Replacement Therapy in Japanese Patients with Chronic Kidney Disease: A Retrospective Cohort Study Using a Claims Database Linked with Specific Health Checkup Results* EHPM. 2021.

eTable 1. Diagnosis codes for chronic kidney disease

Disease	ICD-10 code
Type 1 diabetes mellitus with kidney complications	E102
Type 2 diabetes mellitus with kidney complications	E112
Other specified diabetes mellitus with kidney complications	E132
Unspecified diabetes mellitus with renal complications	E142
Hypertensive chronic kidney disease	I12
Hypertensive heart and chronic kidney disease	I13
Renovascular hypertension	I150
Hypertension secondary to other renal disorders	I151
Acute nephritic syndrome	N00
Rapidly progressive nephritic syndrome	N01
Recurrent and persistent hematuria	N02
Chronic nephritic syndrome	N03
Nephrotic syndrome	N04
Unspecified nephritic syndrome	N05
Isolated proteinuria with specified morphological lesion	N06
Hereditary nephropathy, not elsewhere classified	N07
Glomerular disorders in diseases classified elsewhere	N08
Chronic tubulo-interstitial nephritis	N11
Tubulo-interstitial nephritis, not specified as acute or chronic	N12
Drug- and heavy metal-induced tubulo-interstitial and tubular conditions	N14
Other renal tubulo-interstitial diseases	N15
Renal tubulo-interstitial disorders in diseases classified elsewhere	N16
Chronic kidney disease (CKD)	N18
Unspecified kidney failure	N19
Disorders resulting from impaired renal tubular function	N25
Unspecified contracted kidney	N26
Small kidney of unknown cause	N27
Other disorders of kidney and ureter, not elsewhere classified	N28
Unspecified persistent proteinuria	N391
Unspecified orthostatic proteinuria	N392
Neuropathic heredofamilial amyloidosis	E851
Hemolytic-uremic syndrome	D593
Plasmodium malaria	B52
Renal agenesis and other reduction defects of kidney	Q60
Cystic kidney disease	Q61
Congenital obstructive defects of renal pelvis and congenital malformations of ureter	Q62
Other congenital malformations of kidney	Q63

eTable 2. Codes in relation to cancer and cancer therapy

eTable 2.1. Diagnosis codes for cancer

	ICD-10 code
Neoplasms	C00–D48

ICD-10: International Classification of Diseases-10

eTable 2.2. Therapeutic category codes for cancer therapy

Drugs	Code
Chlorethylamines	4211
Ethyleneimines	4212
Sulfonate esters	4213
Other alkylating agents	4219
Mercaptopurines	4221
Methotrexates	4222
Fluorouracils	4223
Cytosines	4224
Other antimetabolites	4229
Mitomycin C	4231
Chromomycin A3	4232
Actinomycin D	4233
Bleomycin	4234
Anthracycline antibiotics	4235
Neocarzinostatins	4236
Other antibiotics for cancer therapy	4239
Plant extract preparations	4240
Other antitumors	4291
Other unclassified antitumors	4299

eTable 3. Medical procedure codes related to the initiation of renal replacement therapy

	Procedure code	Description
**Dialysis**	B001	Outpatient medical management fees for chronic maintenance dialysis patients
	C102	Medical instruction fees for home self-care peritoneal dialysis
	C102-2	Medical instruction fees for frequent home hemodialysis
	J038	Artificial kidney
	J042	Peritoneal dialysis (continuous ambulatory peritoneal dialysis)
**Renal transplantation**	K780	Cadaveric renal transplantation
	K780-2	Living kidney transplantation

eTable 4. Details of lifestyle behaviors

Lifestyle behavior			Definition
Smoking habits	Current smoking		Having smoked 100 or more cigarettes or smoked for at least six months, and has been smoking over the past month.
Exercise habits	Regular exercise		Exercising to light sweating for over 30 minutes/time, two times weekly, for over a year.
	Regular Walking		Walking or exercising any equivalent amount of physical activity more than 1 hour/day.
	Walking fast		Walking faster than the speed of those around of the same age and sex.
Dietary habits	Frequent skipping breakfast		Skipping breakfast more than three times/week.
	Eating speed	Slow	Eating fast compared to others.
		Normal	
		Fast	
	Eating dinner late		Eating supper two hours before bedtime more than three times/week.
	Frequent late-evening snacking		Eating snacks after supper more than three times/week.
Drinking habits	Alcohol drinking frequency	Rare	Frequency of drinking.
		Occasional	
		Daily	
	Alcohol consumption	<1 glass of sake	Amount of alcohol drinking/day.
		1–2 glasses of sake	
		2–3 glasses of sake	
		≥3 glasses of sake	
Rest and sleep			Taking sufficient rest along with sleep.

eTable 5. Diagnosis codes related to cardio- and cerebrovascular diseases

Disease	ICD-10 code
**Cardiovascular diseases**	
Angina pectoris	I20
Acute myocardial infarction	I21
Subsequent myocardial infarction	I22
Certain current complications following acute myocardial infarction	I23
Other acute ischemic heart diseases	I24
Chronic ischemic heart disease	I25
Pulmonary embolism	I26
Other pulmonary heart diseases	I27
Other diseases of pulmonary vessels	I28
Acute pericarditis	I30
Other diseases of pericardium	I31
Pericarditis in diseases classified elsewhere	I32
Acute and subacute endocarditis	I33
Nonrheumatic mitral valve disorders	I34
Nonrheumatic aortic valve disorders	I35
Nonrheumatic tricuspid valve disorders	I36
Nonrheumatic pulmonary valve disorders	I37
Endocarditis, valve unspecified	I38
Endocarditis and heart valve disorders in diseases classified elsewhere	I39
Acute myocarditis	I40
Myocarditis in diseases classified elsewhere	I41
Cardiomyopathy	I42
Cardiomyopathy in diseases classified elsewhere	I43
Atrioventricular and left bundle-branch block	I44
Other conduction disorders	I45
Cardiac arrest	I46
Paroxysmal tachycardia	I47
Atrial fibrillation and flutter	I48
Other cardiac arrhythmias	I49
Heart failure	I50
Complications and ill-defined descriptions of heart disease	I51
Other heart disorders in diseases classified elsewhere	I52
**Cerebrovascular diseases**	
Nontraumatic subarachnoid hemorrhage	I60
Nontraumatic intracerebral hemorrhage	I61
Other and unspecified nontraumatic intracranial hemorrhage	I62

eTable 6. Therapeutic category codes for drugs on diabetes, hypertension, and dyslipidemia

Drug	Code
**Diabetics**	
Pancreatic hormones	2492
Sulfonylureas	3961
Biguanides	3962
Other antidiabetic agents	3969
**Hypertension**	
Beta blocking agents	2123
Thiazides	2132
Antialdosterones	2133
Chlorbenzene sulfonamides	2135
Other diuretics	2139
Hydralazines	2142
ACE inhibitors	2144
Other antihypertensives	2149
Coronary dilators	2171
Miscellaneous agents (amlodipine besylate and atorvastatin calcium hydrate)	2190
**Dyslipidemia**	
Clofibrates	2183
Other hyperlipidemia agents	2189
Miscellaneous agents (amlodipine besylate and atorvastatin calcium hydrate)	2190

eTable 7. Kidney Disease: Improving Global Outcomes (KDIGO) CKD Classification at baseline

		Initiation of renal replacement therapy	
		(–)	(+)
Number of subjects		149,325	295
eGFR categories	eGFR (ml/min/1.73m^2)		
G1	≥90	6,853 (7.5)	0 (0.0)
G2	60-89	30,732 (33.6)	4 (3.9)
G3a	45-59	51,752 (56.6)	11 (10.8)
G3b	30-44	1,777 (1.9)	14 (13.7)
G4	15-29	243 (0.3)	23 (22.6)
G5	<15	86 (0.09)	50 (49.0)
Missing		57,882	193
Proteinuria categories			
A1	No proteinuria (dipstick −or +/−)	86,770 (59.0)	33 (12.5)
A2	Mild proteinuria (dipstick 1+ or 2+)	58,229 (39.6)	124 (46.8)
A3	Severe proteinuria (dipstick ≥3+)	2,089 (1.4)	108 (40.8)
Missing		2,237	30
Risk stages			
Low		14,493 (15.9)	0 (0)
Moderate		71,947 (78.8)	2 (2.0)
High		3,929 (4.3)	12 (12.1)
Very high		889 (0.97)	85 (85.9)
Missing		58,067	196

Data are presented as the number of subjects (%).

CKD patients were classificated into four risk stages on the basis of eGFR categories (G1 to G5) and proteinuria categories (A1 to A3). Low, G1 or 2 categories and A1 category; Moderate, G3a category and A1 category, or G1 or 2 categories and A2 category; High, G3b category and A1 category, G3a category and A2 category, or G1 or 2 categories and A3 category; Very high, G4 or 5 categories and A1 category, G3b, 4 or 5 categories and A2 category, or G3a, G3b, 4 or 5 categories and A3 category.

eTable 8. Prevalences of missing value in each variable

	Initiation of renal replacement therapy	
Variable	(–)	(+)
Number of subjects	149,325	295
BMI	1,477 (1.0)	11 (3.7)
eGFR	57,882 (38.8)	193 (65.4)
Proteinuria (dipstick)	2,237 (1.5)	30 (10.2)
Current smoking	9,270 (6.2)	27 (9.2)
Regular exercise	23,674 (15.9)	51 (17.3)
Regular walking	30,115 (20.2)	76 (25.8)
Walking fast	29,905 (20.0)	64 (21.7)
Frequent skipping breakfast	23,775 (15.9)	51 (17.3)
Eating speed	32,668 (21.9)	89 (30.2)
Eating dinner late	31,856 (21.3)	88 (29.8)
Frequent late-evening snacking	28,407 (19.0)	74 (25.1)
Alcohol drinking frequency	18,346 (12.3)	44 (14.9)
Alcohol consumption	57,330 (38.4)	132 (44.7)
Sufficient rest along with sleep	27,750 (18.6)	66 (22.4)

BMI: Body Mass Index; eGFR: estimated Glomerular Filtration Rate.
Data are presented as the number of subjects (%).

eTable 9. Baseline characteristics of patients with or without missing of eGFR

Variable	eGFR value status	
	Available value	Missing value
Number of subjects	91,545	58,075
Follow-up time, years, median [IQR]	2.3 [1.2,3.4]	2.3 [1.3,3.4]
Age, years, median [IQR]	52 [46,59]	51 [45,58]
Type of insurance, n (%)		
Insured workers	71,309 (77.9)	48.727 (83.9)
Dependents	20,236 (22.1)	9,348 (16.1)
Female, n (%)	28,040 (30.6)	16,366 (28.2)
BMI, kg/m^2, mean (SD)	23.9 (3.9)	24.2 (4.3)
Proteinuria (1+ or more, dipstick), n (%)	25,918 (28.3)	34,632 (59.6)
Diagnosis of CKD, n (%)	18,972 (20.7)	26,044 (44.8)
Medication for diabetes, n (%)	8,668 (9.5)	9,249 (15.9)
Medication for hypertension, n (%)	26,197 (28.6)	19,735 (34.0)
Medication for hypercholesterolemia, n (%)	18,726 (20.5)	12,299 (21.2)
History of cardiovascular disease, n (%)	12,710 (13.9)	8,976 (15.5)
History of cerebrovascular disease, n (%)	4,841 (5.3)	3,349 (5.8)
Number of check-ups taken, median [IQR]	3 [1,4]	2 [1,4]

BMI: Body Mass Index; CKD: Chronic Kidney Disease; eGFR: estimated Glomerular Filtration Rate; IQR: Interquartile Range; SD: Standard Difference.

Data are presented as the number of subjects (%), median [IQR], or mean (SD).

Missing values were included for calculation of the prevalence of patients who had proteinuria.

eTable 10. Baseline lifestyle behaivors of patients with or without missing of eGFR

Variable	eGFR value status	
	Available value	Missing value
Number of subjects	91,545	58,075
Current smoking, n (%)	20,390 (22.3)	18,176 (31.3)
Regular exercise, n (%)	20,274 (22.2)	12,264 (21.1)
Regular walking, n (%)	26,006 (28.4)	14,110 (24.3)
Walking fast, n (%)	32,999 (36.1)	20,726 (35.7)
Dietary habits, n (%)		
Frequent skipping breakfast	9,013 (9.9)	9,199 (15.8)
Eating speed		
Slow	7,277 (8.0)	3,405 (5.9)
Normal	42,252 (46.2)	26,207 (45.1)
Fast	22,117 (24.2)	15,605 (26.9)
Eating dinner late	20,604 (22.5)	14,991 (25.8)
Frequent late-evening snacking	12,060 (13.2)	7,293 (12.6)
Alcohol drinking frequency, n (%)		
Rare	34,310 (37.5)	20,034 (34.5)
Occasional	24,688 (27.0)	17,017 (29.3)
Daily	21,070 (23.0)	14,111 (24.3)
Sufficient rest along with sleep, n (%)	45,592 (49.8)	29,108 (50.1)

Data are presented as the number of subjects (%).

Missing values were included in the calculation of prevalence for each lifestyle behavior.
